# Subclavian Artery Aneurysm Rupture: A Case Report

**DOI:** 10.7759/cureus.76580

**Published:** 2024-12-29

**Authors:** Joana Castro Vieira, Mafalda Maria Santos, João Vieira Afonso, Mariana Simão de Magalhães, Ana Cristina Teotónio

**Affiliations:** 1 Internal Medicine, Unidade Local de Saúde do Oeste - Hospital Distrital de Caldas da Rainha, Caldas da Rainha, PRT

**Keywords:** atherosclerosis, hemoptysis, intrathoracic aneurysm, medical emergency, saccular subclavian artery aneurysm

## Abstract

Subclavian artery aneurysm is an extremely rare condition with potentially life-threatening complications, including rupture and embolization. This condition is generally the result of atherosclerosis, medial degeneration, trauma, or infection.

We report the case of an 83-year-old man who developed hemoptysis due to the rupture of a saccular aneurysm at the origin of the left subclavian artery. This case highlights the importance of early recognition and emergency management of subclavian artery aneurysms, which, despite their rarity, carry significant morbidity and mortality risks.

## Introduction

Subclavian artery aneurysms are rare conditions, accounting for less than 1% of all peripheral aneurysms [1]. The main causes include atherosclerosis, trauma, and thoracic outlet syndrome, but rarer causes, such as cystic medial necrosis and syphilis, may also occur [2]. These aneurysms are more frequently observed in men aged over 50 [3].

The clinical presentation depends on the location and type of aneurysm. Subclavian artery aneurysms are classified based on their anatomical location as intrathoracic, which are often asymptomatic but may cause symptoms due to compression, and extrathoracic, which most commonly present as a pulsatile mass. Clinical complications can be severe, ranging from compression to spontaneous rupture. Rupture is estimated to occur in about 9% of documented cases with associated mortality of 19% [4].

## Case presentation

We present the case of an 83-year-old Caucasian male patient with a history of dyslipidemia, type 2 diabetes mellitus, and a 40-pack-year history of smoking. The patient was admitted to the emergency department with a sudden onset of dyspnea, cough, and massive hemoptysis. These symptoms started approximately one hour before admission. There was no history of chest trauma or pulmonary tuberculosis.

On physical examination, the patient had a Glasgow Coma Scale score of 15. He presented with mucocutaneous pallor, intense sweating, and abundant hemoptysis. Sinus tachycardia (120 bpm) and fever (38.3 °C) were present. The patient was normotensive, and no difference in blood pressure was noted between the two arms. Pulmonary auscultation revealed decreased breath sounds in the left hemithorax.

**Figure 1:**
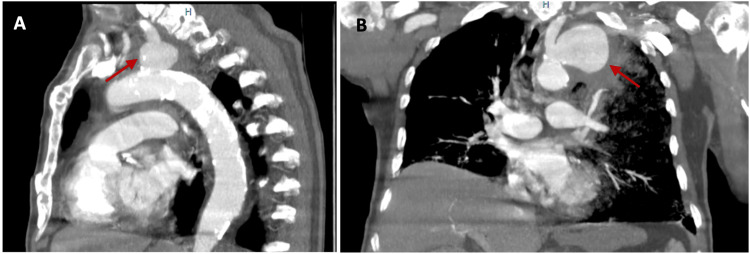
Chest CT angiography: (A) sagittal and (B) coronal images showing the saccular aneurysm at the origin of the subclavian artery (red arrows). CT, computed tomography

**Figure 2:**
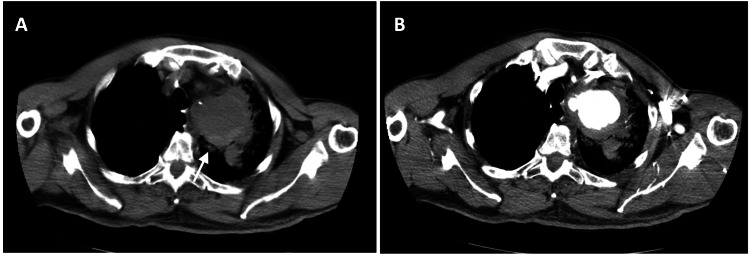
Chest CT angiography on the axial plane: (A) non-contrast acquisition showing hyperdensity suggestive of recent hemorrhage with periaortic and intrapulmonary extension (white arrow); (B) contrast-enhanced axial image revealing intense enhancement in the previously hyperdense areas. CT, computed tomography

**Figure 3:**
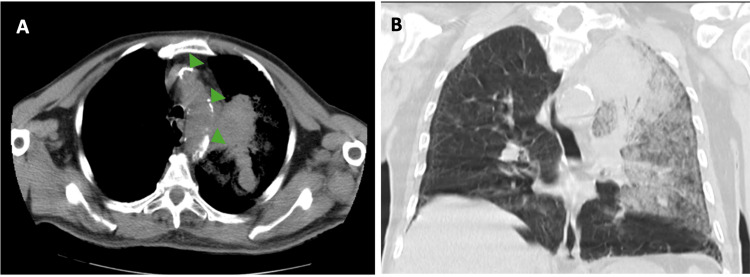
Chest CT angiography: (A) axial view showing calcification of the aorta (green arrowheads); (B) unenhanced coronal view, showing left pulmonary flooding in the context of aneurysmal rupture. CT, computed tomography

Laboratory tests showed a slight elevation of inflammatory markers (Table 1). Serologies for HIV Ag/Ab, Hepatitis B and C, and syphilis were negative, as were the blood cultures. Chest computed tomography (CT) angiography revealed a saccular aneurysm at the origin of the left subclavian artery (Figure 1). The CT scan revealed signs of rupture and erosion at the adjacent pulmonary apex, including hyperdensity, vessel wall disruption, and erosion, which suggested recent rupture and hemorrhage with periaortic and intrapulmonary extension (Figures 2-3). Additionally, diffuse aortic calcification was noted (Figure 3A).

**Table 1:** Laboratory investigations. CBC, complete blood count; WBC, white blood cells;  PT, prothrombin time; INR, international normalized ratio; APTT, activated partial thromboplastin time; LFT, liver function test; AST, aspartate transaminase; ALT, alanine transaminase; LDH, lactase dehydrogenase; KFT, kidney function test; BUN, blood urea nitrogen; Na, sodium; K, potassium; CRP, C-reactive protein; HbA1c, glycated hemoglobin

Test	Observed value	Reference range
CBC		
Hemoglobin (g/dL)	13.3	13.6-18.0
WBC (uL)	15.60 x 10³	4.0 x 10³ to 10.0 x 10³
Platelets (uL)	279 x 10³	140.0 x 10³ to 440.0 x 10³
Coagulation profile		
D-dimers (ng/mL)	2180	<500
PT (seconds)	16.2	9.0-13.0
INR	1.3	0.8-1.2
APTT (seconds)	34	25.1-36.5
LFT		
Total bilirubin (mg/dL)	0.72	0.20-1.20
AST (U/L)	14	5-34
ALT (U/L)	8	0-55
LDH (U/L)	146	125-220
KFT		
BUN (mg/dL)	30	18-55
Serum creatinine (mg/dL)	0.72	0.70-1.30
Na (mEq/L)	130	136-145
K (mEq/L)	3.9	3.5-5.1
Additional tests		
CRP (mg/dL)	18.4	< 0.5
Procalcitonin (ng/mL)	0.09	<0.5
HbA1c (%)	7.8	4.0-6.0

The patient was initially managed at a district hospital without specialized vascular expertise and was urgently referred to the regional vascular surgery team. Despite limited resources, the patient remained stable, allowing for transfer to a specialized unit for definitive treatment.

The patient underwent an emergent procedure for the exclusion of a saccular aneurysm at the origin of the left subclavian artery. The procedure included thoracic endovascular aortic repair (TEVAR) via right femoral access, embolization with coils of the prevertebral segment of the left subclavian artery via humeral access, and a left carotid-subclavian bypass. The surgery was performed under balanced general anesthesia. During the procedure, he required norepinephrine support at a maximum dose of 8 mcg/minute to maintain hemodynamic stability. Coagulopathy was observed with an intraoperative international normalized ratio (INR) of 1.95, necessitating the administration of 400 mL of fresh frozen plasma. Despite these complications, lactate levels remained normal, and urine output was maintained at 0.6 mL/kg/hour, reflecting adequate end-organ perfusion.

The patient was transferred to the Intensive Care Unit intubated, under mechanical ventilation, and continued to require hemodynamic support and metabolic control. After seven days in intensive care, he showed clinical improvement, was weaned from mechanical ventilation, and was transferred to the vascular surgery ward. He remained stable and was eventually discharged in an improved condition. Unfortunately, the patient passed away a few days after discharge, despite the intervention for the ruptured subclavian artery aneurysm.

## Discussion

Subclavian artery aneurysms, although rare, represent an important clinical entity due to the high risk of complications. They can be classified as extrathoracic, frequently identified by the presence of a pulsatile nodule in the supraclavicular fossa, and intrathoracic, generally associated with compressive or ischemic symptoms [4,5].

Intrathoracic aneurysms are typically atherosclerotic and can cause hemoptysis when eroding to adjacent structures, such as the pulmonary apex. Compression of the recurrent laryngeal nerve can lead to hoarseness, while tracheal compression, although rare, can cause dyspnea [6]. In this case, the most likely etiology of the subclavian artery aneurysm is atherosclerosis, supported by the patient's multiple risk factors (dyslipidemia, diabetes mellitus, and heavy smoking) and the presence of aortic calcifications observed on the chest CT scan. The aneurysm was located in the proximal part of the subclavian artery, a region typically associated with aneurysms of atherosclerotic cause [6].

Chest CT angiography is essential in the diagnosis and therapeutic planning, allowing the localization and characterization of the aneurysm, as well as identifying signs of complications such as rupture and thrombosis.

Rupture is a severe and often fatal complication; therefore, surgical or endovascular correction should always be considered, particularly in symptomatic cases or those at high risk of rupture [7]. This is highlighted in a comprehensive review of 394 cases by Andersen et al., which reported rupture in 9% of patients. These findings reinforce the critical importance of timely intervention, especially in symptomatic or high-risk aneurysms, such as the one described in this report. Modern TEVAR-based endovascular techniques have significantly expanded repair options for subclavian artery aneurysms, offering excellent results and enabling less invasive procedures for both the aneurysms themselves and associated thoracic aortic pathologies in many patients. The majority of these aneurysms can now be repaired using a TEVAR-based approach without the need for sternotomy or thoracotomy. These advancements not only reduce procedural morbidity but also broaden the scope of treatment for patients previously considered high risk for open surgical interventions [3,4].

The patient, in this case, had a ruptured aneurysm, necessitating urgent investigation and intervention. Comorbidities, along with limited resources and specialist availability at a district hospital, posed significant management challenges. However, the patient remained stable, enabling transfer to a specialized unit for surgical intervention.

## Conclusions

Although rare, subclavian artery aneurysms can lead to severe complications such as rupture and embolization. Rupture presents as an emergency with severe chest pain, shock, or loss of consciousness, requiring immediate surgical or endovascular intervention.

This case highlights the importance of early recognition, accurate diagnosis, and timely treatment to prevent rupture and minimize fatal outcomes.
